# Effect of Modulated Electromyostimulation on the Motor System of Elderly Neurological Patients. Pilot Study of Russian Currents Also Known as Kotz Currents

**DOI:** 10.3389/fphys.2022.921434

**Published:** 2022-07-18

**Authors:** Liubov Amirova, Maria Avdeeva, Nikita Shishkin, Anna Gudkova, Alla Guekht, Elena Tomilovskaya

**Affiliations:** ^1^ Laboratory of Gravitational Physiology of the Sensorimotor System, Institute of Biomedical Problems of the Russian Academy of Sciences, Moscow, Russia; ^2^ Consultative and Diagnostic Department, Solovyov Scientific and Practical Psychoneurological Center of the Moscow Department of Health, Moscow, Russia

**Keywords:** electromyostimulation, Russian currents, timed up and go test, neurological patients, postural stability

## Abstract

In this brief report, we present preliminary findings from a study of the use of electromyostimulation (EMS) in neurological patients. Assuming the approach to be sufficiently effective, we decided to investigate the motor system of elderly neurological patients before and after a course of Russian currents EMS, which were developed for Soviet athletes and cosmonauts. To this point, 19 patients—EMS (*n* = 11) and control (*n* = 8)—have successfully completed the study. The study included patients aged 60–90 years with confirmed walking and balance disorders with a history of chronic cerebral ischemia. Patients in the experimental group underwent a course of modulated EMS of the hip and shin muscles from 3 to 9 procedures. Preliminary results of the study showed good patient acceptance of EMS. After the course, the EMS group showed a significant improvement from baseline in the Tinetti Test (+1.4 points, *p* = 0.0045), Rivermead Mobility Index (+0.5 points, *p* = 0.0022), and Timed Up and Go Test (−1.2 s, *p* = 0.0053). There was also a significant improvement in balance quality of 8.6% (*p* = 0.04). Shin muscle strength, although trending positively, did not change significantly. There was also no change in hip and shin muscles’ tone. No significant changes were observed in the control group in the same tests. It can be concluded that stimulation of the hip and shin muscles with Russian (Kotz) currents has a positive effect on the motor system of elderly neurological patients. Significant effects with a course of short duration indicate that this EMS regimen is promising.

## 1 Introduction

Older age is accompanied by a gradual decline in body functions, including decreased physical activity. Age-related changes are known to affect the motor areas of the brain, and as a consequence, posture, gait and fine motor skills suffer (Seidler et al., 2010), which in turn may mediate an even greater decline in motor activity. Skeletal muscles are particularly susceptible to the effects of aging, and with age they steadily lose function and mass. The decline in functional performance is associated with a general decline in muscle integrity as fibrosis and fat accumulation replace functional contractile tissue, as well as the loss of the fastest and most powerful fibers (Scicchitano et al., 2009; Vinciguerra et al., 2010). Prolonged course of exercises is known to counteract muscle weakness: it increases protein synthesis, metabolism and satellite cell number, stimulates appetite, increases IGF-1 expression levels and capillary bed density (Paffenbarger et al., 1994; Kern et al., 2014). However, it is not always possible to maintain sufficient level of physical activity, and electromyostimulation (EMS) can be an alternative to intensive physical exercise.

The main advantage of the EMS approach to rehabilitation is the wide coverage of patients with a variety of medical histories (Jones et al., 2016). The use of EMS is possible even in cases where physical activity is difficult or impossible due to cardiovascular (Arenja et al., 2021; Poltavskaya et al., 2021), pulmonary (Zanotti et al., 2003) and other conditions (Arija-Blazquez et al., 2014; Nussbaum et al., 2017). Adherence to bed rest has been shown to reduce muscle strength by 5–7% (Pisot et al., 2008). EMS has also been shown not only to increase muscle strength but also to reduce lower limb spasticity after stroke (Moon et al., 2017), and a 1-week course of stimulation of the quadriceps femoris and peroneus longus muscles of both legs results in an increase in hip and shin circumferences (Gerovasili et al., 2009). Thus, EMS is a promising treatment/countermeasure modality (Arenja et al., 2021).

One of the methodological approaches to EMS is the so-called Russian currents developed by Y.M. Kotz's group for Soviet athletes (Kotz and Hvilon, 1971), and it was later adapted for cosmonauts. It is important to note that the method is a unique development that differs from its counterparts (NOT high voltage pulsed current or whole-body EMS, etc.). The essence of this type of stimulation is the modulation of medium frequency current (2.0–5 kHz) by lower frequencies (10–100 Hz). Due to these stimulation parameters, the evoked muscle contractions are as close as possible to physiological ones, which reduce the discomfort of the procedure, patient fatigue, and also has an anesthetic effect in itself (Rampazo and Liebano, 2022). It is also suggested that such a configuration of the electrical signal may help to achieve visible results in a shorter time than with unmodulated currents (Ward and Shkuratova, 2002).

The aim of this work was to assess the motor system using 1) standard clinical questionnaires and tests, measurements of 2) postural stability and 3) muscle tone in elderly patients before and after EMS course with Russian currents. We hypothesised that electromyostimulation using Russian currents, also called Kotz currents, could improve the overall motor system in neurological patients in 10 treatments or less.

## 2 Methods

### 2.1 Participants

To this point, 19 people have successfully completed the study. Two more people dropped out of the study: one for the medical reasons described below, the other for failing to attend the final examination. The patients were initially divided into two subgroups: EMS (*n* = 11) and control (*n* = 8). Although the patients of both sexes were considered as subjects in this examination, for one reason or another only woman participated in both subgroups so far. Details of the study participants, including height, weight and cognitive scores on the MMSE scale, are presented in Supplementary Table S1 of the supplementary materials.

Patients were admitted to the hospital with complaints of dizziness and unsteadiness when walking. After an initial examination by a neurologist, meeting the study criteria (see below) and signing voluntary informed consent, patients were randomly allocated into either the EMC or control groups. Participation in the study did not exclude the performance of physiotherapeutic procedures (magnetotherapy, darsonvalization, electrosleep therapy) and therapeutic exercises (vascular gymnastics and exercises for spinal osteochondrosis). Given the inpatient profile, most patients received standard vascular and metabolic therapy, as well as antidepressants and neuroleptics. The groups were homogeneous in terms of the treatment used.

Inclusion criteria for the study were elderly and old age (60–90 years according to WHO) with confirmed test results of impaired walking and balance in patients with a history of chronic cerebral ischemia.

Patients with high spasticity (3 or more by the modified Ashworth scale), atrial fibrillation, infectious processes, impaired pain sensitivity, epilepsy, lower limb joint endoprosthetics, low scores (less than 24 b, corresponding to mild dementia) on the MMSE scale were excluded from the study.

### 2.2 Electromyostimulation

The duration of electrostimulation was designed for a 2-week inpatient stay (two courses of 5 days each) in addition to medication treatment. However, due to coronavirus restrictions, between 3 and 9 EMS procedures were performed (median 7). Data on the number of EMS treatments are shown in Supplementary Table S2 of the supplementary materials. The procedure was always performed in the first half of the day. Patients were not warmed up in any way before the EMS procedure.

Patients in the control group did not receive electromyostimulation sessions. However, they received standard treatment.

**FIGURE 1:**
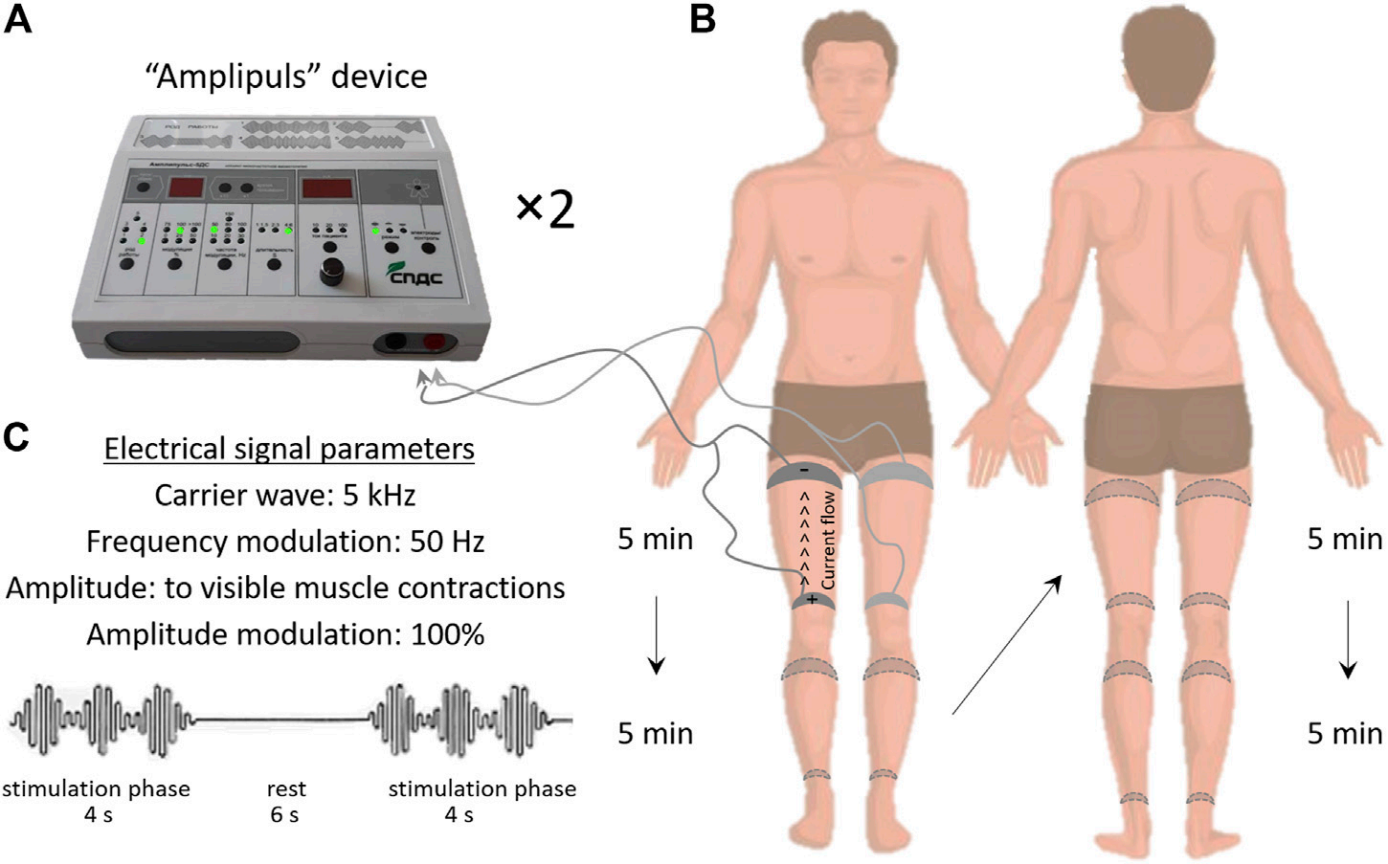
Electromyostimulation protocol. General view of the Amplipulse-5DS stimulator **(A)**, electrical signal parameters **(B)**, electrode arrangement and order of stimulation of the lower limb segments **(C)**.

Patients in the EMS group underwent lower limb stimulation in sinusoidal modulated current mode (not to be confused with high voltage pulsed current) using two single-channel Amplipulse-5DS stimulators (Russia) (Figure 1A). Carrying frequency of sinusoidal oscillations was 5 kHz, modulation frequency—50 Hz (Figure 1B). Amplitude of stimulation was set according to its peak tolerance by the patient. The range of amplitudes was from 10 to 40 mA, but the group average mean was 22–25 mA (Supplementary Figure S1 of supplementary materials). Stimulation and rest periods amounted 4 and 6 s, respectively (Figure 1B). The total duration of one session was 20 min, 5 min each for stimulation of the anterior and posterior hip muscles (mm. semitendinosus, biceps femoris, quadriceps femoris) and shin (mm. triceps surae, tibialis anterior) (Figure 1C). The timing of the stimulation was chosen according to the protocol used by the cosmonauts. Wet electrodes (5 × 15 cm) of conductive rubber were used.

### 2.3 Study Design

Patients were interviewed and a battery of tests was performed twice: on admission to hospital and after the last EMS session. A battery of tests was conducted in the sequence below.

*The Tinetti Scale* (Scura and Munakomi, 2022) and *the Rivermead Mobility Index* (Williams, 2011) were used to determine the degree of activity of daily living.

The Timed Up and Go (2010) Test was performed according to a standard protocol (Kear et al., 2017). The patient had to get up from a chair, walk for 3 m, turn around, go back and sit down. The time over the distance was recorded.

*Postural stability* was assessed using the BioMera stabilography platform (BioMera LLC, Russia). The patients stood on the platform for 1 minute with their eyes open, and then another minute with their eyes closed. The fluctuations of the centre of pressure (CoP) were recorded. Path length (L), velocity of CoP movement (V) and statokinesiogram area (S) were analyzed. The Equilibrium Score (EQ), a dimensionless parameter assessing the ability to maintain equilibrium, was also calculated according to the formula: EquiScore = [1-(P-Psway)/12.5°]*100, where P-Psway is the maximum oscillation of the center of pressure in the sagittal plane, 12.5° is assumed to be the limit of stability for a normal individual (Chaudhry et al., 2004). To obtain the displacement of the center of gravity from the stabilogram, it was filtered with a 2nd order Butterworth high-pass filter that cut off frequencies above 0.85 Hz. The center of gravity was considered to be a point located at 55% of the height of the subject (Shishkin et al., 2019).

*Muscle tone* was assessed using MyotonPRO device (MyotonLTD, Estonia). The device applies mechanical impulses of stable strength and duration, and registers damped harmonic oscillations, from which viscoelastic properties of the studied tissue are calculated using a special mathematical algorithm (Schneider et al., 2015). The viscoelastic properties of mm. soleus, gastrocnemius lat and med, tibialis anterior, semitendinosus, biceps femoris, rectus femoris, and vastus lat were examined. During this examination, subjects were laying in prone or supine positions. In order to standardize the position of the lower limbs, rollers were placed under the knee and ankle joints. Standard parameters (Stiffness, Frequency, and Creep) were analyzed, the calculation procedure of which can be found elsewhere (Schneider et al., 2015).

*The Strength test* for the shin muscles was performed in the supine position using a specially designed tensometric pedal. The patient's leading leg was fixed in a position with the ankle, knee and hip joint angles of 90°. The patient made three attempts of peak plantar flexion—maximal foot pressure on the pedal (engaging the shin muscles, but not the hip muscles), the best result was recorded. The maximal voluntary force (MVF) developed by the lower leg muscles was analyzed.

### 2.4 Statistics

A two-way RM ANOVA with Sidak's posterior criterion was applied to determine significant statistical differences in the Tinetti, Rivermead, Up and Go, Strength, and muscle tone tests. The Mixed-effects model (REML), using the posterior Sidak test, was used to perform statistical analysis of stabilographic parameters (L, V, S). The Mann Whitney test was applied to determine statistical differences in the percentage difference in EQ between the EMS and control groups. The significance level is α = 0.05. All data are presented as Mean ± SEM.

## 3 Results

### 3.1 Effects of Electromyostimulation on Patients’ General Well-Being and Tolerance

The study found that the hip and shin electrostimulation procedure was well tolerated by all age groups of patients and no significant side effects were reported. A patient with giant Baker's cysts (anamnestic) presented with moderate pain in the knee of the affected limb during EMS was excluded from the study. Another patient had a minor hemorrhoidal bleeding once after the first treatment, which was caused by the drug combination. Thereafter, no bleeding was observed after correcting the therapy and the patient completed the course of therapy. Some patients experienced short-term moderate muscle pain, as after intensive physical activity for 1–2 days which was probably due to excessively high amplitude during stimulation.

Slight subjective improvement was noticed after 3-4 sessions. After a course of 8-9 sessions all the patients noticed a subjective improvement of postural stability, gait and ability to climb stairs more easily. It is also worth noting that the procedures were interesting and positive for the patients.

### 3.2 Questionnaires and Tests

**FIGURE 2:**
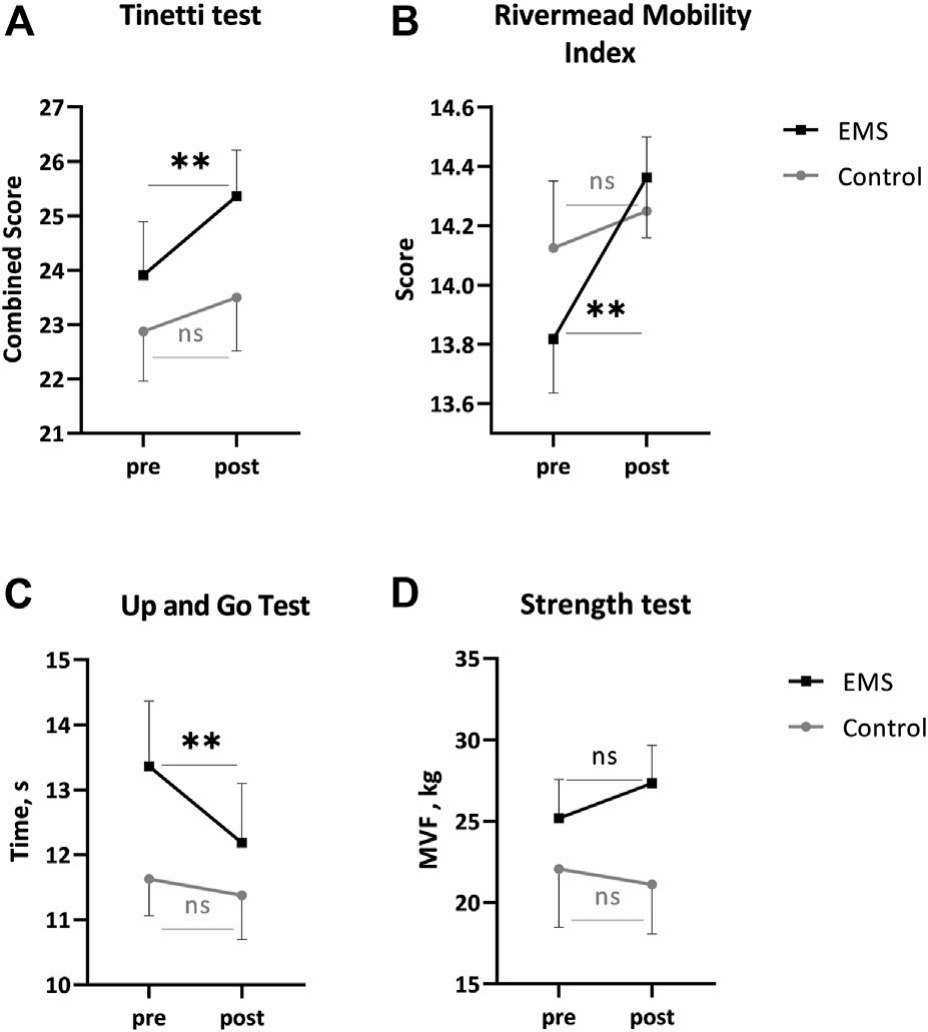
Main indicators from the Tinetti test. **(A)**, Rivermead Mobility Index **(B)**, Up and Go **(C)**, Strength **(D)**. Mean ± SEM. **p < 0.002 pre vs. post.

The pre-study, groups scored 23.9 ± 0.9 (EMS) and 22.8 ± 0.9 (control) on the Tinetti test (Figure 2A). Post examination, the EMS group showed a significant improvement (+1.4 points, *p* = 0.0045), in contrast to the control group, where no such improvement was observed (+0.6 points, *p* = 0.4).

The Rivermead Mobility Index showed a similar pattern of change (Figure 2B). The initial pattern (13.8 ± 0.1 in EMS and 14.1 ± 0.1 in control) changed to a significant increase post-EMS (+0.5 points, *p* = 0.0022). No significant changes were found in the control group (+0.1 points, *p* = 0.7).

The TUG test showed the following values before the study: 13.3 ± 1.0 s in the EMS and 11.6 ± 0.5 s in the control groups (Figure 2C). The EMS patients accelerated the distance run by 1.2 s (*p* = 0.0053) post-study. No significant changes were found in the control group (− 0.25 s, *p* = 0.7).

The maximal voluntary force was 25.1 ± 2.3 kg and 22.0 ± 3.5 kg in the EMS and control groups, respectively (Figure 2D), pre-study. No significant changes were recorded for MVF in two groups post-study.

### 3.3 Postural Stability

Post EMS course, patients visually improved postural stability, while no changes were observed in the control group.

**FIGURE 3:**
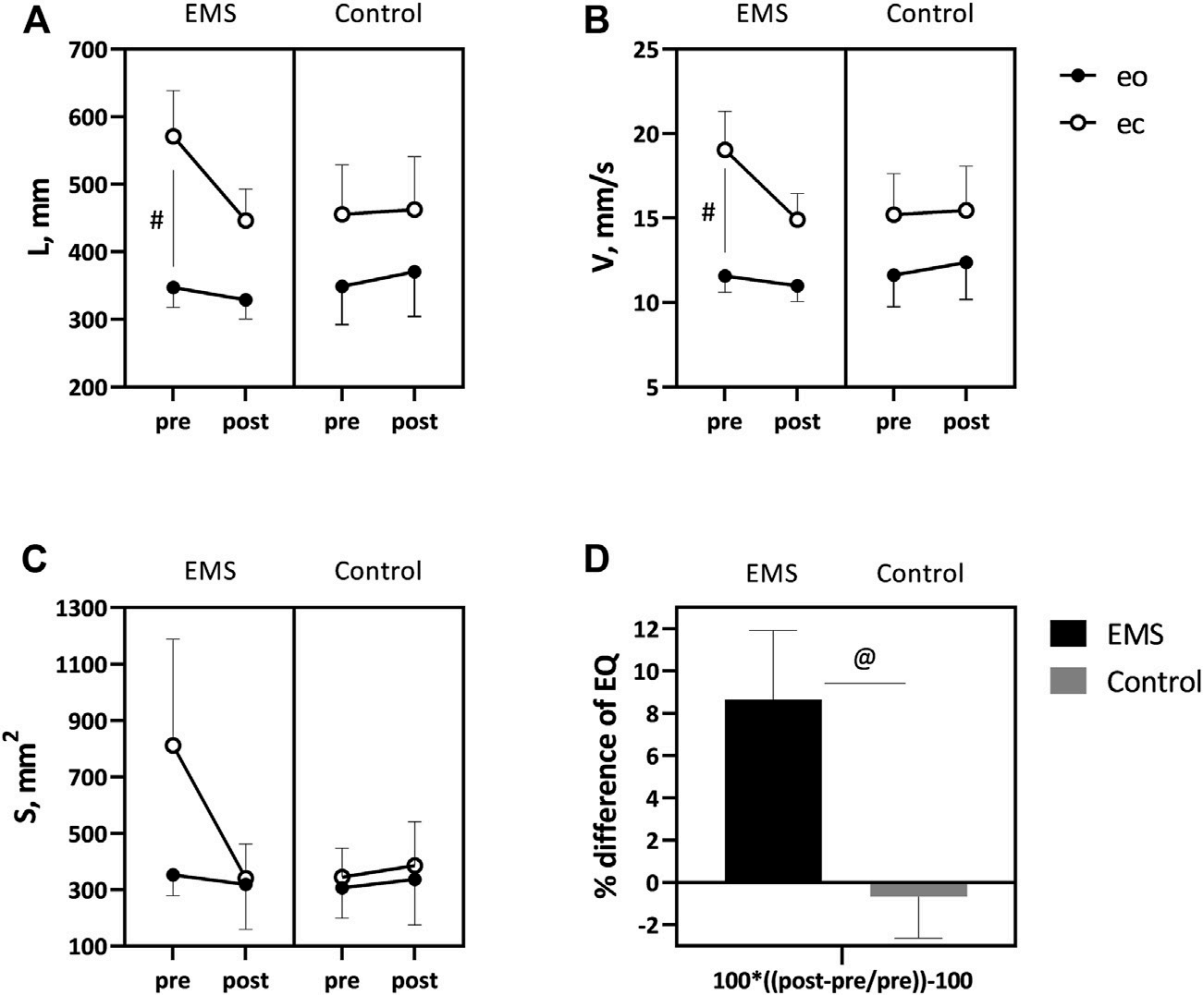
Comparative data for length **(A)** and velocity **(B)** of the CoP and area of the statokinesiogram **(C)** in eyes open (shaded icons) and closed (not shaded icons), and also change in EQ **(D)** in the two patient groups. Mean ± SEM. # −*p* < 0.05 eyes open vs. eyes closed.at −*p* < 0.05 EMS vs. control.

L was 347.1 ± 29.2 mm in pre-study in the EMS group with eyes open, increasing significantly to 571.0 ± 67.9 mm with eyes closed (Figure 3A). In post-study, there was a slight downward trend in L in both open and closed eyes. In the control group, however, L with the open eyes was 349.1 ± 56.7 mm, increasing slightly to 455.7 ± 73.3 mm when the eyes were closed. No significant changes in L were observed after the study in the control group.

Similar trends were shown by V. Baseline V values in the EMS group amounted 11.5 ± 0.9 mm/s and 19.0 ± 2.2 mm/s for eyes open and closed, respectively (Figure 3B). Post-study, V was not significantly different from baseline values. In the control group, pre-study V values were 11.6 ± 1.8 mm/s for eyes open and 15.2 ± 2.4 mm/s for eyes closed. No significant changes in V were observed at the post-study period.

S in the EMS group was 352.8 ± 73.3 mm^2^ in eyes open and 812.0 ± 377.1 mm^2^ in eyes closed (Figure 3C). Post-EMS application, S showed a decreasing trend, which, however, was not significant. In the control group, S was 306.8 ± 107.5 mm^2^ in eyes open and 345.0 ± 102.9 mm^2^ in eyes closed. At the post-study, S was slightly higher than baseline values, 336.7 ± 161.0 mm^2^ in eyes open and 385.6 ± 156.5 mm^2^ in eyes closed.

The EQ, which is an integral indicator of an individual's postural stability, was calculated from the data obtained. Pre-study, the EQ was 80.0 ± 10.6 in the EMS group and 85.2 ± 5.8 in the control group out of a possible 100 points (Figure 3D). Post the EMS course, balance quality improved by 8.6% in the stimulation group (*p* = 0.04). No changes were registered in the control group.

### 3.4 Muscle Tone

Comparative data on the muscle tone are shown in Table 1. In the vast majority of cases, no significant changes in the studied parameters were found.

**TABLE 1:** Comparative table of the main parameters of skeletal muscle tone properties in two groups of patients.

	*Stiffness*		*Frequency*		*Creep*	
	*Pre*	*Post*	*Pre*	*Post*	*Pre*	*Post*
m. semitendinosus						
EMS	235,1	229,5	12,9	12,8	1,7	1,7
Control	249,6	236,3	14,2	13,0*	1,5	1,6
m. biceps femoris						
EMS	286,4	276,4	15,4	15,2	1,3	1,4
Control	278,9	280,9	15,7	15,2	1,4	1,4
m. gastrocnemius lat						
EMS	282,4	290,8	14,5	15,5	1,5	1,4
Control	280,7	294,8	14,8	14,8	1,4	1,4
m. gastrocnemius med						
EMS	273,2	267,5	15,5	13,1	1,6	1,6
Control	273,1	273,6	12,9	13,1	1,5	1,5
m. rectus femoris						
EMS	270,6	277,6	13,4	13,9	1,5	1,4
Control	257,9	256,4	12,6	12,5	1,6	**1,7** **^#^**
m. soleus lat						
EMS	333,2	350,9	16,7	17,7	1,2	1,1
Control	**405,7** **^#^**	391,6	**20,1** **^#^**	19,7	**0,9** **^#^**	**0,9** **^#^**
m. soleus med						
EMS	341,4	335,1	16,4	16,6	1,1	1,2
Control	371,9	372,3	18,0	17,3	1,0	1,0
m. tibialis anterior						
EMS	387,5	393,9	19,4	19,5	0,9	0,9
Control	399,2	384,8	19,1	18,0	1,0	1,0
m. vastus lat						
EMS	279,8	281,7	13,9	14,7	1,5	1,4
Control	259,2	253,9	12,8	13,4	1,7	1,5

# −*p* < 0.05 EMS, vs. Control. *− *p* < 0.05 pre vs. post.

## 4 Discussion

Thus, preliminary studies involving an initial small sample of neurological patients after EMS treatment with Russian currents support our hypothesis by 1) improvement in daily motor skills, a decrease in distance time and a trend towards increased muscle strength and 2) stabilisation of vertical stance. However, there is no change in muscle tone in patients after EMS 3). No significant changes in all parameters were recorded in the control group.

The application of EMS in patients with diseases of different genesis is not new and is actively used in medicine (Nussbaum et al., 2017; Silva et al., 2020). In particular, much of this research has been devoted to the treatment and countermeasure for elderly people (Evangelista et al., 2021), those with muscle weakness (Jones et al., 2016) or those in hypodynamic conditions (Wageck et al., 2014). In this study, we used Russian currents regimens analogous to use for cosmonauts, which should give the greatest effectiveness in a short period of time. We assumed that each patient would be able to receive 9–10 EMS procedures, but the coronavirus restrictions imposed reduced the number of sessions. We did not expect that the 6.3 procedures that our participants received on average would have such a significant effect in almost all tests and examinations performed. Previously it had been shown that 6 EMS procedures over 4 weeks had a positive effect on the 6-min walking test and muscle strength in patients, but in addition to the stimulation sessions the participants performed physical exercises (squats, lunges, biceps curl, chest press, butterfly reverse, reverse lunges, standing diagonal crunches, etc), which does not allow to make a direct conclusion on the results. In the vast majority of cases, however, there is a long duration of courses, ranging from several weeks to a year (Kemmler et al., 2018).

One of the most informative tests to assess the functional status of older adults is the Up and Go test (Freiberger et al., 2013). In a study involving elderly volunteers, it was shown that a 9-week EMS (24 training sessions) improved the time to pass the TUG test by 16.4% (Kern et al., 2014). In our study, a similar improvement was 9.0%, but it was achieved in a shorter time.

In the Nishikawa study, lower extremity stimulation for 12 weeks in elderly patients with dementia improved muscle strength (*p* = 0.008) and postural stability (*p* = 0.007), in contrast to the control group where these characteristics worsened (Nishikawa et al., 2021). In our study, an improvement in postural stability in patients after EMS treatment was reliably recorded at an earlier time point. However, in contrast to Nishikawa's work, no significant increase in muscle strength was recorded in our study, but a clear upward trend in MVFwas observed in the EMS group. It is likely that a significant increase in strength requires a bigger amount and/or duration of EMS sessions.

A factor that we did not take into account in this study was life satisfaction and general emotional mood while in hospital. We also did not record patients’ daily motor activity. There is evidence that the psycho-emotional state of elderly patients may have an effect on their motor function (Beheydt et al., 2014). However, we believe that since the main difference between the groups was the use of EMC, it is more likely that this fact can explain the results.

It also needs to be mentioned that for some baseline parameters, the EMS and control groups were slightly different, although there were no significant differences. For example, TUG time was 1.7 s better in the control group (13.3 s EMS vs. 11.6 s control). According to the classification (Bischoff et al., 2003), community-dwelling elderly women should be able to complete the TUG test in 12 s or less, which correlates with the control group. The TUG test values of the group with EMC, although significantly improved after a course of Russian currents, still averaged more than 12 s. However, according to the classification of the American College of Rheumatology (2010), test results of 10–20 s are considered normal for older adults.

It is also important to note that this article is a preliminary report of the results. The small number of participants so far allows us to draw intermediate conclusions about the beneficial effects of short courses of modulated EMS. We plan to increase the number of participants in each group as well as to add another group with sham stimulation to offset the effects of the procedure itself, such as the application of electrodes, the extra attention of the nursing staff and the feeling of current. Research into the effects of EMS on the motor performance of patients with stance and walking disorders will continue and the final results will be published at a later date.

## 5 Conclusion

It can be concluded that stimulation of the hip and shin muscles with Russian (Kotz) currents has a positive effect on the motor system of neurological patients of advanced age. Significant effects with a course of short duration indicate the promise of this EMS treatment regimen. However, the preliminary character of the data requires further research to form significant conclusions.

## Data Availability

The raw data supporting the conclusions of this article will be made available by the authors, without undue reservation.
