# Erratum: Effect of modulated electromyostimulation on the motor system of elderly neurological patients. Pilot study of Russian currents also known as Kotz currents

**DOI:** 10.3389/fphys.2022.1032040

**Published:** 2022-09-28

**Authors:** Frontiers Production Office

**Affiliations:** Frontiers Media SA, Lausanne, Switzerland

**Keywords:** electromyostimulation, Russian currents, timed up and go test, neurological patients, postural stability

Due to a production error, there was a mistake in affiliation 2. Instead of “Laboratory of Gravitational Physiology of Sensorimotor System, Institute of Biomedical Problems of Russian Academy of Sciences, Moscow, Russia,” it should be “Consultative and Diagnostic Department, Solovyov Scientific and Practical Psychoneurological Center of the Moscow Department of Health, Moscow, Russia.” Three authors—Maria Avdeeva, Anna Gudkova, and Alla Guekht—were assigned the wrong affiliation. The author list should therefore appear as follows:

Liubov Amirova^1^*^†^, Maria Avdeeva^2†^, Nikita Shishkin^1^, Anna Gudkova^2^, Alla Guekht^2^ and Elena Tomilovskaya^1^

The publisher apologizes for this mistake. The original version of this article has been updated.

