# Supplementary material for: Effect of Modulated Electromyostimulation on the Motor System of Elderly Neurological Patients. Pilot Study of Russian Currents Also Known as Kotz Currents
Source: Front Physiol. 2022 Jul 18;13:921434. doi: 10.3389/fphys.2022.921434 (PMC9339608; doi:10.3389/fphys.2022.921434)
Supplement: Supplementary file 1 [file Table1.DOCX]

Supplementary Material

Effect of modulated electromyostimulation (Russian currents) on the motor sphere of elderly neurological patients

Liubov Amirova^*^†, Maria Avdeeva†^2^, Nikita Shishkin^1^, Anna Gudkova^2^, Alla Guekht^2^, Elena Tomilovskaya ^1^

^1^Laboratory of Gravitational Physiology of Sensorimotor System, Institute of Biomedical Problems of Russian Academy of Sciences, Moscow, Russia

^2^Consultative and Diagnostic Department, Solovyov Scientific and Practical Psychoneurological Center of the Moscow Department of Health, Moscow, Russia

**Table I.** Data from patients who took part in the survey

| Subgroups | Patient No. | Full age | Height, cm | Weight, kg | MMSE cognitive assessment (max. 30 points) |
| --- | --- | --- | --- | --- | --- |
| EMS | 1 | 65 | 162 | 77,8 | 29 |
| EMS | 2 | 69 | 167 | 83,7 | 29 |
| EMS | 3 | 76 | 148 | 63,6 | 28 |
| EMS | 4 | 64 | 170 | 95,2 | 29 |
| EMS | 5 | 79 | 160 | 80 | 29 |
| EMS | 6 | 74 | 163 | 61,6 | 28 |
| EMS | 7 | 80 | 168 | 66,8 | 28 |
| EMS | 8 | 74 | 165 | 76 | 28 |
| EMS | 9 | 80 | 169 | 72,4 | 25 |
| EMS | 10 | 81 | 153 | 76,1 | 27 |
| EMS | 11 | 62 | 175 | 60,2 | 30 |
| ***Average*** | | ***73,1*** | ***163,6*** | ***73,9*** | ***28,2*** |
| ***Standard deviation*** | | ***7,0*** | ***7,8*** | ***10,5*** | ***1,3*** |
| control | 12 | 64 | 149 | 62,9 | 28 |
| control | 13 | 64 | 156 | 84,6 | 29 |
| control | 14 | 75 | 149 | 42,6 | 26 |
| control | 15 | 73 | 156 | 75 | 26 |
| control | 16 | 82 | 160 | 72,5 | 26 |
| control | 17 | 80 | 160 | 75,4 | 27 |
| control | 18 | 80 | 150 | 79 | 26 |
| control | 19 | 72 | 152 | 77 | 28 |
| ***Average*** | | ***73,8*** | ***154,0*** | ***71,1*** | ***27,0*** |
| ***Standard deviation*** | | ***7,0*** | ***4,6*** | ***13,1*** | ***1,2*** |

**Table II.** Data on the number of EMC procedures in patients

| Patient No. | Number of EMS procedures |
| --- | --- |
| 1 | 7 |
| 2 | 7 |
| 4 | 6 |
| 5 | 9 |
| 6 | 3 |
| 7 | 8 |
| 8 | 6 |
| 9 | 3 |
| 10 | 5 |
| 11 | 8 |
| 12 | 7 |
| ***Average*** | ***6,3*** |
| ***Standard deviation*** | ***2,0*** |
| ***25% percentile*** | ***5*** |
| ***median*** | ***7*** |
| ***75% percentile*** | ***8*** |

**Figure I.** Average stimulation amplitudes. Mean±range
